# The role of traditional healers in the diagnosis and management of Burkitt lymphoma in Cameroon: understanding the challenges and moving forward

**DOI:** 10.1186/s12906-017-1719-y

**Published:** 2017-04-11

**Authors:** Glenn M. Afungchwi, Peter B. Hesseling, Elena J. Ladas

**Affiliations:** 1 Banso Baptist Hospital, Bui Division, Northwest region, Kumbo, Republic of Cameroon; 2 Department of paediatrics and child health, Tygerberg children’s Hospital, Stellenbosch University, Cape Town, South Africa; 3 Division of Pediatric Hematology/Oncology/Stem Cell Transplant and Institute of Human Nutrition, Columbia University Medical Centre, 3959 Broadway, New York, New York CHN 10-06A USA

**Keywords:** Burkitt lymphoma, Traditional medicine, Supportive care, Traditional healer, Cameroon

## Abstract

**Background:**

Burkittlymphoma(BL) is the most common childhood cancer in Cameroon with a reported incidence of 3 per 100,000 children under 15 years in the Northwest region. Treatment at three Baptist mission hospitals has a recorded cure rate of over 50%. Traditional medicine(TM) is recognized by the national health system, but its scope is undefined and entraps children with BL. The aim of this study was to investigate the attitudes and practices of parents and traditional healers (TH) towards TM in children with BL in order to develop recommendations for an integrative approach and improved access to life-saving treatment for children with BL.

**Methods:**

This is a descriptive case series of children diagnosed with BL treated at Banso, Mbingo, and Mutengene Baptist Hospitals between 2003 and 2014. A questionnaire was used to obtain the following information: demographic information, religion, the rate of use of TM, reasons why guardians chose to use TM, the diagnoses made by the TH, treatment offered, and the type of payment requested, based on the accounts of patient caregivers. Data was analyzed using Center for Disease Control Epi Info 7.

**Results:**

Three hundred eighty-seven questionnaires were completed by parents/guardians. 55% had consulted a TH, of whom 76.1% consulted the TH as first choice. Common diagnoses provided by TH included liver problem, abscess, witchcraft, poison, hernia, side pain, mushroom in the belly and toothache. Methods of management included massage, cuts, concoctions, and incantations. The fee for these services included chickens, farm tools, and cash ranging from 200FCFA (0.4USD) to 100,000FCFA(200USD). The choice of TM was based on accessibility, failed clinic/hospital attendance, recommendation of relatives, and belief in TM.

**Conclusions:**

TH are involved in BL management in Cameroon. TH are ignorant about BL, resulting in non-referral, and thus delay in diagnosis and treatment. Collaboration with TH could reduce late diagnosis and improve cure rates of BL and other childhood cancers.

## Background

Childhood cancer constitutes a major public health problem in Cameroon [1]. Incidence rates in Africa are not readily available due to the limited availability of cancer registries [2]; however, several global studies performed in low- and middle-income countries (LMICs) have documented a rise in its incidence over the past decade. This is attributed to several factors, but is partly due to improved education and training of medical personnel in the identification of childhood cancer. Burkitt lymphoma(BL) is the most common childhood cancer in Cameroon, with a reported incidence rate of 3 per 100,000 children below age 15 years in the Northwest region [3]. BL classically presents with a facial and or abdominal swelling. Previously, there was only one national paediatric cancer treatment centre in Yaounde; however, since 2003, paediatric cancer treatment services have been established at three Baptist mission hospitals in rural Northwest and Southwest Cameroon. Diagnosis of BL remains a challenge in Cameroon with the scarcity oncologist and pathology services. Diagnosis in the northwest region is accomplished by cytology of fine needle aspirations, Bone marrow aspirations, and cerebrospinal fluid, as well as on the basis of abdominal ultrasound [4]. An event-free survival rate of 60% has been reported for BL at these rural centres [4], with a long-term survival of over 50% [5].

Studies have demonstrated that up to 90% of children with cancer use some form of traditional medicine (TM); with the use of TM significantly higher in LMICs compared to high-income countries [6, 7]. Surveys have also found that the use of TM has been associated with delays in access to conventional treatment as well as abandonment of therapy. These challenges have been even more pronounced in Africa where indigenous practices are deeply embedded in the culture and way of life. In Cameroon, TM as a practice has evolved naturally over many generations. It is an attractive source of health care, which consumes an estimated 7% of household health care expenditure [8]. Given the significant advances in the treatment of childhood cancer, increased attention is warranted to ensure that children are offered effective treatments for cancer while simultaneously recognizing the role of TM.

The World Health Organization (WHO) in the Alma Ata Primary Health Care Declaration recognized the importance of TM in providing primary health care [9]. This recognition has been moderated with the provision of specific guidelines for assessment of safe practices applicable to traditional medicine practice [10]. The practice of traditional medicine is officially recognized in Cameroon, with an office of traditional medicine at the ministry of public health and its incorporation in all levels of the health care system [11]. Civil administrators are expected to allow the practice of traditional medicine in their health localities. In 1993 a national association was formed for the promotion of traditional medicine, offering registration and licensing to traditional healers (THs) who show certain levels of competence [12].

The practice of traditional medicine in Cameroon remains largely undefined and includes children with (undiagnosed) cancer, including BL. There is limited data from Africa as a whole on TM and pediatric oncology despite being home to a large percentage of children with cancer simultaneously being a region with a deep and complex history of TM. The objective of this study was to investigate the current diagnostic and management methods used by THs for children with BL.

## Methods

This is a descriptive case series of children diagnosed with BL treated at Banso, Mbingo, and Mutengene Baptist Hospitals between 2003 and 2014. The inclusion criterion was children between the ages of 0and 15 years with a confirmed cytological diagnosis or clinical diagnosis of BL, experiencing a response to induction treatment, and without any contrary cytology report [4, 5]. A questionnaire was used to obtain the following information: demographic information, religion, the rate of use of TM, reasons why guardians chose to use TM, the diagnoses made by the TH, treatment offered, and the type of payment requested, based on the accounts of patient caregivers. Diagnosis of BL was confirmed from participants’ medical charts.

The primary respondents were the parents and other caregivers who brought the children to the hospital for treatment. The questionnaire wasapplied after counselling of parents about their child’s disease, treatment, expected course, cost and need for follow up. Participation was entirely optional, with no effects on the treatment and other supportive care offered. The study was approved by the institutional review board and informed consent was obtained.

The questionnaire was read and explained to respondents by a research assistant nurse at each centre. These nurses were all involved in the care of the child. Despite a literacy rate of 75% for persons above 15 years in Cameroon [13], most of the patients enrolled in the centres were from rural settings and predominantly from farming families with a relatively lower level of literacy.

### Statistical analysis

Demographic characteristics were summarized as median, range, mean, standard deviation for continuous variables, and as counts and percentages for nominal variables. Statistics were generated using Center for Disease Control Epi Info™.

## Results

The 387 questionnaires completed represent 42% of all patients treated, with 222 (57.4%) males, 164 (42.4%) females, and one not recorded (0.2%). The median age was 8 (range1-17 years), with a mean of 7.9 years(SD +/− 2.4). Respondents were primarily Christian (*N* = 251;68.9%) followed by Muslims (*N* = 116; 30%), and not specified (*N* = 20;5%). Two hundreds and thirteen (55%) respondents had consulted a TH before admission, 162 of whom (76.1%) had consulted the TH as their first choice before attending a health centre or hospital and the remaining 51 (23.9%) after prior consultation at a local conventional health centre or hospital. The rate of use of TM was 54.6% amongst Christians and 53.4% amongst Muslims.

Participants reported several reasons for seeking out a TH for their child (Table 1). For the majority of participants, ineffective hospital treatment or family beliefs were the primary reasons for seeking treatment with TH. Advice from the community was influential in choosing TH as well as beliefs in the theories of TH such as witchcraft. Importantly, we found that participants sought out TH for symptoms that are usually not well-controlled in conventional health care facilities in Cameroon such as pain management.

**Table 1:** Reasons for consulting traditional healers

REASON	FREQUENCY	PERCENTAGE FREQUENCY
Previous visits to a clinic/hospital did not help	51^a^	24%
Family belief and preference for Traditional Medicine (TM)	51	24%
Advice from neighbours or family	31	15%
Thought it was witch craft	19	9%
Had no money	13	6%
Unknown to respondent	9	4%
Thought it was a strange(bad) disease that only a traditional healer (TH) can treat	7	3%
Was the nearest source of pain relief for the child	7	3%
Knows that a TH is good at treating side pain	5	2%
Knowledge that ‘boh’ is usually treated by a TH	3	1%
Knows that a TH is good at treating boil/abscess	2	1%
Knows a TH is good at spleen problems	2	1%
Thought liver problems cannot be treated in the hospital	1	<1%
The TH saw child and offered to help	1	<1%
The TH is renowned for fracture treatment	1	<1%

^a^surgery in two cases

**Fig. 1:**
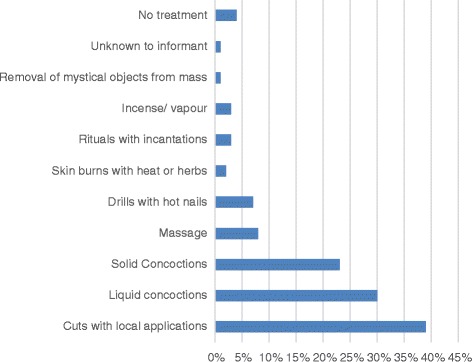
Treatment methods used by traditional healers to treat Burkitt lymphoma

**Fig. 2:**
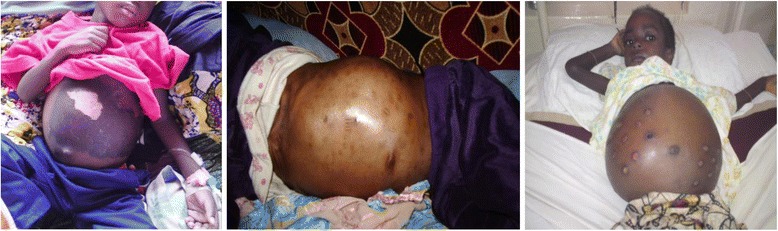
*Treatment methods used by traditional healers to treat Burkitt lymphoma.* *Left to Right*: Skin burn with corrosive herbs; skin cuts over region of tumour; drills into tumour through the skin by use of heated nails

TH provided a wide-variety of causes of the child’s illness (Table 2). Witchcraft was the most frequent explanation given for the disease, followed by disease of the spleen or liver and abscess or boil. The disease was described as cancer in a few cases. In some the patients were managed with no explanation given for the disease. A variety of interventions were used to treat the illness. The methods included skin cuts, burns or drills, with local application of herbal pastes on cut wounds, and the ingestion of solid and liquid concoctions (Fig. 1 and Fig. 2).

**Table 2:** The various explanations for Burkitt’s lymphoma by traditional healers

EXPLANATION	FREQUENCY	PERCENTAGE FREQUENCY
Witch craft	35	16%
Spleen diseases(enlargement)	35	16%
No explanation	27	13%
Abscess/ Boil	13	6%
Side pain	10	5%
Belly bite	9	4%
Liver disease	8	4%
Informant does not know	8	4%
Hernia	6	3%
Toothache/ dental problem	6	3%
Poison	6	3%
Bladder stone	5	2%
Abdominal disease	4	2%
Leg problem (paralysis)	4	2%
Growth/cancer	4	2%
‘Boh’(Mushroom)	3	1%
Worms	3	1%
Sinusitis	3	1%
Frog in the abdomen	2	1%
Faecal mass	2	1%
Malaria	2	1%
Kwashiokor	2	1%
Mumps	2	1%
Strong sick	1	0.5%
Goitre	1	0.5%
Rheumatism	1	0.5%
Yellow fever	1	0.5%
Overeating	1	0.5%
Lung disease	1	0.5%
Growth pains	1	0.5%
Child abuse	1	0.5%
Double umbilicus	1	0.5%
Child’s shadow not in body	1	0.5%
Dirt in the belly	1	0.5%
Injury after fall	1	0.5%
Palpitations	1	0.5%
Moving object in the body	1	0.5%

Finally, our survey evaluated the form of payment for the TM treatment (Table 3). The majority of participants (54%) paid money in exchange for the services provided by a TM healer while others exchanged items for treatment in the barter system. In some cases, there was no charge for the TH’s services or payment was deferred awaiting recovery.

**Table 3:** Payment Methods for the Services of Traditional Healers

Method of payment	Number	Frequency
Money (USD 0.4 – USD 200)	114	54%
Barter system^a^	84	40%
No charge	50	24%
Unknown to informant	5	2%
Payment deferred till recovery	2	1%
Any token	1	0.5%

^a^The Barter system included the provision of fowls, palm oil, salt, palm wine, cutlass, and cooking pots in exchange for treatment

## Discussion

To the authors’ knowledge, this is the first report on the use of TM among children with cancer in Cameroon. We found that the majority (55%) of guardians/parents consulted a TH for their child’s disease, which is within previously reported rates of 15% to 95% [14–16]. Our results confirm the description of TM by the WHO as a common, but underestimated, treatment method [9]. We also found that respondents consulted a TH because they did not have money, or because it was the nearest source of help. Similar findings have been reported in other studies in Cameroon [12] and elsewhere in Africa [15]. This supports the WHO’s description of TM as the most available and affordable treatment option in sub-Saharan Africa [17]. Importantly, our results underscore the need for educational initiatives aimed at the conventional and TM community.

Delays in the diagnosis and initiation of treatment of childhood cancer is a significant factor impacting survival among children located in LMICs. Hesseling et al. reported 84% of children with BL in Cameroon had St. Jude’s Stage III or IV disease at time of diagnosis [4]. Consultation with THs may be a factor in delayed diagnosis of BL. Our survey found that 76% of users of TM sought treatment with a TH before attending a conventional health centre or hospital, which is a figure in line with other reports in Sub-Saharan Africa [17]. In Ngaoundere, the Adamawa region of Cameroon, TH is the most available form of health care for patients outside urban settings due to the paucity of health centres and hospitals [12]. Our survey found that the remaining participants (24%) who visited a TH did so after consulting a local health centre or hospital, illustrating a significant need for more knowledge on BL amongst the health care professionals in these health care units.

The reasons reported for choosing TM reflects a high level of belief in TM as part of the overall culture of the population, given the variety of ethnic groups whom each have a unique natural medicinal heritage [18]. The authors could not find any outcome reports for children with BL treated by TM, on the contrary there is report of 60% event free survival for children with BL treated in three childhood cancer treatment centers in the Northwest and Southwest regions of Cameroon [4]. Health practitioners sometimes fail to suspect BLbecause of insufficient knowledge about the disease. A survey in three of the seven divisions of Northwest Cameroon revealed that 51% of rural nurses do not know about BL. They make incorrect diagnoses, provide the wrong treatment, and fail to refer patients to cancer treatment centers. Therefore, there is an urgent need for trained health care personnel to provide sensitization to health workers at the community level about the diagnosis and available treatment of BL patients. Such sensitization efforts have been made in the Northwest region of Cameroon, and ongoing efforts are being made to provide this knowledge to practicing nurses, and nurses in training. The inclusion of childhood cancers in the training curricula for nurses nationwide will further help address this problem.

Nearly 10% of participants reported that witch craft was the cause of disease and 8% believed that witchcraft was the cause of the cancer [15]. This finding supports previous investigations that beliefs in TM theories are strongly linked to use of TM [19]. We found that diagnoses made by TH are based on superstition, attribution to commonly known diseases with even distant similarities in physical appearance, or suspicion of a mystical affliction. This denotes a lack of knowledge about BL amongst TH in the region, which can be assumed a general situation for all childhood cancers given that BL is the most common childhood cancer reported in the country [1]. Collectively, this finding suggests that childhood cancer awareness is limited amongst health care providers in Cameroon, including TH and underscores the need to educate TH on childhood cancer presentations and the availability of curative care. Such a training for TH was done on a large scale by the South African Childhood Cancer Parents Organization (CHOC) [20]. Additionally, widespread Childhood Cancer awareness campaigns by the Cameroon Baptist Convention Childhood cancer program are underway. The goal of these programs isto educate communities on the early signs of childhood cancer, by use of group lectures and the distribution of brochures and flyers.

We found that treatment modalities were not too different in other developing countries [6, 14–16], but cuts and drills are unique to Cameroon [6]. TM in Cameroon is a practice that is learned by inheritance, apprenticeship, or as a gift from the ‘spirits’. The diagnostics and treatments of TH are guided by the way they learn their trade. The most common methods are those that harness the rich biodiversity of the local forests, grasslands, and maritime geographies [21]. The plurality of methods used in traditional medicine constitutes a major setback to its credibility, and facilitates the proliferation of charlatans [12]. The need to sensitize TH about BL is clear, given the fact that they form a legal part of the Cameroon health care system, and offer care to about 80% of the population at various points in time [12]. These TH need to be included in the surveillance for pediatric cancers, and be educated on algorithms for referral of suspected cases.

Our study is limited by the cross-sectional design and as such is subject to its limitations. The survey was administered by staff nurses who had other patient care duties resulting in inconsistent availability for administering the survey as well as limited time to collect all demographics of interest. A developing regional initiative is addressing this in a comprehensive manner. There is also the possibility of biased response since these nurses were members of the patient care teams. Due to limited staffing, we were unable to examine survival differences between patients who used TM and those who did not use TM. However, this is an important point for future investigation. Additionally, this study does not investigate the association between socioeconomic status and the use of TM. However, it is observed that the lack of money is the fourth most common reason why patients of children with BL seek medical care from TH.

## Conclusion

In conclusion, THs constitute an important part of the health care system in Cameroon. Despite the non-standardized nature of their diagnostic and interventional practices and charges, they arguably have a genuine concern for the health of the populations they serve. This study convincingly shows a significant knowledge deficit among THabout the presentation of BL. This is only one ramification of the overall low level of awareness on pediatric cancers in the general population [22]. We believe that respectful collaboration, and education of TH on the early warning signs of cancer, and the availability of good curative and palliative care, will increase the number of children with BL who are suspected in he communities and referred to specialized centers to be diagnosed and adequately treated. Such collaboration with THs has created a tremendous difference in care for patients with HIV/AIDS in Africa [23], and must continue to be explored to improve survival rates for children with cancer.

## Abbreviations

AIDS: Acquired immunodeficiency syndrome
BL: Burkitt lymphoma
CDC: Center for disease control
CHOC: Childhood cancer parents’ organization
FCFA: Franc communauté financière africaine
HIV: Human immunodeficiency virus
LMIC: Low-middle-income countries
TH: Traditional healer
TM: Traditional medicine
USD: United States dollars
WHO: World Health Organization
